# Context-dependent female mate choice maintains variation in male sexual activity

**DOI:** 10.1098/rsos.170303

**Published:** 2017-07-12

**Authors:** Carolin Sommer-Trembo, Martin Plath, Jakob Gismann, Claudia Helfrich, David Bierbach

**Affiliations:** 1 College of Animal Science and Technology, Northwest A&F University, Yangling 712100, People's Republic of China; 2 Department of Ecology and Evolution, J. W. Goethe University Frankfurt, Max-von-Laue-Straße 13, 60438 Frankfurt am Main, Germany; 3 Department of Biology and Ecology of Fishes, Leibniz-Institute of Freshwater Ecology and Inland Fisheries, Müggelseedamm 310, 12587 Berlin, Germany

**Keywords:** sexual selection, male mating activity, poeciliids, computer animations

## Abstract

The existence of individual variation in males' motivation to mate remains a conundrum as directional selection should favour high mating frequencies. Balancing selection resulting from (context-dependent) female mate choice could contribute to the maintenance of this behavioural polymorphism. In dichotomous choice tests, mosquitofish (*Gambusia holbrooki*) females preferred virtual males showing intermediate mating frequencies, reflecting females' tendencies to avoid harassment by highly sexually active males. When tested in the presence of a female shoal—which protects females from male harassment—focal females showed significantly stronger preferences for high sexual activity. A trade-off between (indirect) benefits and (direct) costs of mating with sexually active males probably explains context-dependent female mate choice, as costs depend on the social environment in which females choose their mates. No preference was observed when we tested virgin females, suggesting that the behavioural pattern described here is part of the learned behavioural repertoire of *G. holbrooki* females.

## 1. Background

In species in which male investment into individual offspring is low, males should be under directional selection to maximize mating activity, assuming that high mating frequencies increase the number of offspring males will father [1]. Still, pronounced variation of male mating activity in natural populations is observed [2–4]. Much of this variation can be ascribed to competition among males resulting in unequal mating opportunities [5]. Also, it can reflect costs associated with mating, as some males carry parasites [6] or are otherwise physiologically challenged [7]. While the latter studies suggest that male mating activity could be an honest indicator of males' current condition [8,9], there is increasing evidence that male mating behaviour differs consistently among individuals [10,11] even with ample mating opportunities, thus reflecting behavioural profiles rather than plastic responses to environmental and/or social factors (see electronic supplementary material, S1 for Eastern mosquitofish, *Gambusia holbrooki*). These observations raise the question of how intrinsic differences in male sexual activity can be maintained in a population.

Using *G. holbrooki* as our model organism, we tested an as-yet unexplored hypothesis, that male sexual activity may not directly predict their mating success because females reject the most sexually active (most harassing) males depending on social context. *Gambusia holbrooki* has a coercive mating system [12,13] and rejection of highly sexually active males could be a result of costs arising from male sexual harassment, as reported for an array of species [14–16] including *G. holbrooki* [17,18] and other poeciliid fishes [19,20]. In this case, females should evaluate male sexual activity [21] in addition to phenotypic characteristics like colour ornaments [22] or body size [23] as a mate choice criterion.

Assuming that females gain indirect benefits from mating with males exhibiting high sexual activity (a trait their sons may inherit [24]) but simultaneously incur costs from sexual harassment, we predicted that females would show a preference for intermediate male sexual activity, resulting in a nonlinear (stabilizing) preference function. To test this idea, we used binary association preference tests in which we presented focal females with computer animations showing virtual stimulus males that differed only in numbers of sexual approaches towards a virtual female mating partner.

Moreover, *G. holbrooki* females form all-female shoals to protect themselves from male harassment [17,18,25], just like many other fishes form shoals to evade predators [26]. This prompted the hypothesis that females rate male attractiveness differently when they are alone or in a shoal. Specifically, females should exhibit a preference for (rather than aversion of) highly sexually active males in proximity of a protective shoal. We tested this idea with a modified experimental set-up in which the focal female exerted mate choice in the presence of a shoal consisting of three conspecific females. Finally, we asked whether the female preference for male sexual activity—and potential context-specific adjustments of this preference—are innate behavioural features of the species or must be learned through sexual experience [27]. To this end, we contrasted the behaviour of sexually experienced and inexperienced (virgin) females.

## 2. Methods

### 2.1. Study organism and maintenance of test subjects

Eastern mosquitofish (*G. holbrooki*) have a widespread natural range of occurrence in fresh waters of the eastern USA, but have been introduced worldwide to control mosquito larvae and the diseases they transmit [13]. *Gambusia holbrooki* is a livebearing species with internal fertilization. Mating behaviour does not involve courtship, and many mating attempts (called gonopodial thrusts) are coercive [12,28], even though the success of coercive mating attempts in poeciliid fishes appears to be low [29,30]. A frequently observed premating behaviour of non-courting poeciliids is ‘nipping behaviour’ during which males touch the female's gonopore with their snout [21]. Female behaviour (staying close to or swimming away from a male) seems to influence the outcome of forced inseminations [31], underlining the importance of female mate choice in this mating system.

We used fish of a laboratory stock (maintained in our laboratory since 2013) originating from fish collected in Florida. Fish were maintained in mixed-sex stock tanks (200 l, except virgin females, see below) that were equipped with filters, natural gravel and various aquatic plants and stones for shelter. Fish were held at a constant temperature of 24°C under a 12 L : 12 D regime and were fed twice a day with commercially available flake food (TetraMin®), frozen chironomid larvae and *Artemia* sp. To maintain water quality, we exchanged half of the water every two weeks and replaced it by aged tap water.

To obtain virgin females, we raised offspring in separate tanks under conditions as described above. Males were identified by their developing gonopodium [32] and were separated from females before reaching sexual maturity (electronic supplementary material, S2).

### 2.2. Experimental set-up

We tested female responses to males showing different levels of sexual activity. Additional experimentation in our laboratory found numbers of sexual behaviours to range between 0.7 and 3.4 behaviours per minute when ample mating opportunities were provided (one male cohabited with three females; see electronic supplementary material, S1), which is in line with previous studies on this species [17,33]. However, we decided to roughly double the frequencies for low, medium and high sexual activity in the animations for the following reasons: male *G. holbrooki* typically switch between phases of burst sexual activity, during which they show incessant pursuit of females and frequent gonopodial thrusting, and phases of low or no sexual activity (C.S.-T., M.P., J.G., C.H., D.B. 2013–2017, personal observation). During phases of peak sexual activity (which often last no longer than a few seconds), females are exposed to elevated levels of male harassment, which is neglected when averaging male sexual activity over a much longer observation phase. Moreover, in our experiment quantifying variation in male mating activity, we used groups of familiar individuals (electronic supplementary material, S1). Poeciliid males, however, show elevated mating frequencies after brief social isolation or when they encounter a novel female [34–37]—both of which are likely to happen regularly under natural conditions. We, therefore, defined 1.5 sexual interactions per minute as low sexual activity, 3 as medium and 6 as high sexual activity. These frequencies elicited female responses in a pilot study (not shown).

Two days prior to the mate choice tests, we transferred all focal females to different maintenance tanks (80 × 40 × 30 cm), in which they were held in groups of 10–15 individuals. Except for the absence of males, conditions were the same as in the mixed-sex stock tanks. We tested three cohorts of focal females that differed in sexual experience and the social context in which they were tested: (i) sexually experienced females (i.e. adult females from mixed-sex stock tanks) were tested alone (*n* = 15), (ii) sexually experienced adult females were tested with a shoal consisting of three familiar females present (*n* = 15), and (iii) adult virgin females were tested alone (*n* = 18). All test subjects were tested successively for their preferences for different nipping frequencies. In each test run, we presented one of three types of animations (each of them showing a different nipping frequency) on one monitor, while another monitor presented the same animation pair without any sexual behaviour (control). With this binary dichotomous design, we prevented mating preferences from being masked by mere social attraction, as *Gambusia* females tend to form shoals [13,26,38].

The test tank (80 × 30 × 30 cm) was filled with aged tap water to a height of 10 cm (electronic supplementary material, S3). We visually divided the tank into three zones: two lateral preference zones (both 20 cm in length) close to the screens (FUJITSU Display B22 W-5) that showed the stimulus animations, and a neutral zone in the middle (40 cm) [21]. To initiate a trial, we gently transferred a female into a transparent Plexiglas cylinder (11 cm diameter) in the middle of the neutral zone. At this time, both screens were set to show a grey background that matched the grey cardboard with which all sides of the experimental tank were covered (except for an 8 × 20 cm gap on both smaller sides through which the animations were presented). After 1 min of acclimation, we started playback of both animations and the female could observe them for 3 min from its central position. Afterwards, we gently released the female from the cylinder and allowed it to swim freely for 5 min and choose to associate with either type of animation. A camera recorded the female, and we later used the resulting videos to determine how much time the female spent in either of the two association zones. Association times in similar dichotomous mate choice tests have been demonstrated to provide information about female mating preferences in related species [39–41]. To control for side bias, we repeated the procedure with changed side-assignments and summed up females' times near each animation type.

We placed the female back into the cylinder and gave it 2 min for acclimation before we presented the next set of animations. The female thus completed all three preference tests before we measured its standard length (SL) and transferred it back into its original stock tank. We randomized the order of the three animation pairs as well as the side on which the control was shown during the first trial.

In the treatment testing sexually experienced females in the presence of a female shoal, the focal female was accompanied by three familiar individuals (from the same stock) throughout the whole series of preference tests. The shoal was kept in a transparent Plexiglas cylinder (18 cm diameter) which was placed in the back part of the neutral zone. After the preference tests, we measured SL of the shoal mates (29.4 ± 0.6 mm) and returned them to their maintenance tank. We exchanged females used to compose the shoals between trials.

### 2.3. Video animations

Using animated stimuli is a way to standardize behavioural experiments, increases their repeatability and provides robust internal controls (as only the trait of interest will be manipulated while providing otherwise identical stimuli) and has become a powerful tool to study mate choice [42]. Video animations have been successfully applied in behavioural studies on *Gambusia* spp. and other poeciliids [21,43–46].

For the animations, we took photos of 10 males and five females from the same population in lateral view (Canon EOS 600D, © Canon Inc.). We cut out the fish silhouette from the background of the photos and standardized them by adjusting an average body size (SL, males: 21.8 mm, females: 36.0 mm) using Adobe® Photoshop CS5.

To generate animations, we used Adobe® Flash professional CS6 with a screen resolution of 800 × 600 pixels. Each animation showed a female and a male (swimming 1 cm underneath, and slightly behind the female), both of which were moving horizontally from one side of the screen to the other within 10 s. The pair disappeared for 2 s, invisibly changed direction and then moved back in the opposite direction. During the constant movement, we simulated sexual interactions in a way that the male moved upwards to the female, touching her genital pore with his snout (‘nipping’ behaviour; electronic supplementary material, video). We thus created three types of animations with different levels of sexual activity. We decided to use ‘nipping’ behaviour because it is the most visible mating behaviour in non-courting poeciliid species and can be observed frequently [21]. Furthermore, it is easy to animate and poeciliid females respond to animations showing this type of behaviour [21].

To avoid pseudo-replication [42], we created 10 different sets of animations, and each focal female was later presented with one of these sets. We used the same male and female photos for control animations, in which they were shown swimming horizontally from one side of the screen to the other and back without any sexual behaviour. Hence, each focal female saw the same male phenotype in the control and sexual activity animations, differing only in the frequency of animated sexual behaviour.

### 2.4. Statistical analyses

We calculated focal females' strength of preference (SOP) for the sexually active male versus control male as: time spent with sexually active male/time spent near both animation types. SOP-values served as the dependent variable in a linear mixed model (LMM) with ‘focal female ID’ as a random factor. ‘Male sexual activity’ (low, medium and high) and ‘treatment’ (three cohorts of focal females) as well as their interaction term were included as fixed factors. ‘Focal female SL’ was initially included as a covariate but removed from the model as it did not have a significant effect (*F*_1,134 _= 0.60, *p* = 0.44). For post hoc evaluation, a significant preference (SOP > 0.5) or avoidance (SOP < 0.5) was inferred from 95% CIs of the estimated marginal means not overlapping 0.5. Similarly, we inferred significant differences in SOP for different male sexual activity levels within each female cohort when 95% CIs did not overlap (electronic supplementary material, S5). Moreover, we tested if the three cohorts of focal females would differ in total association times (sum of times spent near both types of animations). We compared total association times between the three cohorts using a one-way ANOVA.

All statistical analyses were run in SPSS v. 23.0. Assumptions of normal error distribution and homoscedasticity (as required for the analytical models we used) were met in case of all dependent variables.

## 3. Results

**Figure 1.**
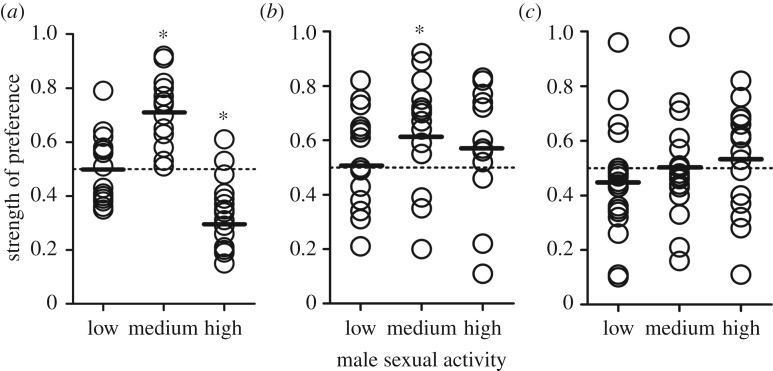
Female preferences for male sexual activity. SOP-values, calculated from association times in binary choice tests (see Methods) of (*a*) sexually experienced *G. holbrooki* females when tested alone, (*b*) sexually experienced females when tested in the presence of a protective shoal of conspecific females and (*c*) single, virgin females. Focal females could choose between computer animations showing a virtual male that exhibited low, medium and high sexual activity (1.5, 3 and 6 pre-copulatory behaviours per minute) and another animation showing a male that exhibited no sexual behaviour. No preference resulted in SOP = 0.5 (dotted line), while values greater than 0.5 indicate attraction, and values less than 0.5 aversive responses towards the sexually active stimulus male. Solid lines represent means of the respective cohort/treatment. Statistical significance (*) was inferred from 95% CIs of estimated marginal means not overlapping SOP = 0.5.

To assess how females evaluate variation in male sexual activity during mate choice, we presented individual focal females with computer-animated males showing three different levels of male sexual activity (*low*: 1.5, *medium*: 3 and *high*: 6 sexual behaviours per minute). Differences in female preference functions among the three cohorts of focal females (sexually experienced females tested alone, sexually experienced females tested in a group and virgin females tested alone) were reflected in our LMM by a significant interaction term of ‘treatment × male sexual activity’ (*F*_4,135 _= 5.77, *p* < 0.001; main effects, treatment: *F*_2,135 _= 2.84, *p* = 0.060; male sexual activity: *F*_2,135 _= 8.52, *p* < 0.001). Sexually experienced females that were tested alone exhibited indiscriminate responses towards males with low sexual activity (i.e. SOP-scores close to 0.5), a preference for males with intermediate sexual activity (SOP greater than 0.5) and aversive behaviour when presented with males showing high sexual activity (SOP < 0.5). This resulted in a bell-shaped preference function (figure 1*a*).

When sexually experienced focal females were tested in the presence of a protective shoal, they showed different responses, with slightly weaker preferences for intermediate sexual activity (which still elicited a positive response, i.e. SOP > 0.5; figure 1*b*). Notably, female responses to males with high sexual activity were significantly different from those observed when females were tested without a shoal present (this difference was evidenced by non-overlapping 95% confidence intervals of estimated marginal means between both cohorts of females; see electronic supplementary material, table S5). In this test situation, females were attracted (SOP > 0.5) to the animation showing males with high sexual activity.

Finally, sexually inexperienced (virgin) females did not show any pattern of attraction or aversion in response to the three types of animations (all SOP-values close to 0.5; figure 1*c*).

Total time spent in both preference zones (= total association time) differed among cohorts (one-way ANOVA: *F*_2,47 _= 32.96, *p* < 0.001) and was highest in virgins (mean ± s.e.: 330 ± 26 s), followed by sexually experienced females (294 ± 13 s). Experienced females tested in a group showed the lowest total association times (112 ± 15 s), probably because they spent more time in the neutral zone associating with the female group. This does, however, not necessarily affect *relative* times spent near the different animations.

## 4. Discussion

Theory predicts directional selection to favour high intrinsic male mating activity [1,5], especially when male investment into individual offspring is low, like in coercive mating systems, as seen in *G. holbrooki*. Nevertheless, *G. holbrooki* males show pronounced and consistent individual variation in sexual activity (electronic supplementary material, S1). In our study, we identified one mechanism that could contribute to the maintenance of the observed variation in male sexual activity. We found sexually experienced females to prefer males showing medium sexual activity and to avoid highly sexually active males when tested alone. However, this preference changed, and highly sexually active males became more attractive when females were tested with a group of conspecific females. Sexually naive (virgin) females did not show any preference related to male sexual activity.

If consistent differences in male sexual activity have a heritable component, they are potentially subject to selection, including intersexual selection. We tested the hypothesis that females alter their preference for highly sexually active mating partners depending on the current social context. Single *G. holbrooki* females preferred intermediately sexually active males but avoided high levels of sexual activity. These results suggest balancing (rather than directional) selection acting on male sexual activity levels through female mate choice. We argue that this pattern results from a trade-off between indirect benefits and direct costs associated with highly sexually active males. High male sexual activity probably increases mating rates and, therefore, the number of offspring males can father—a trait that sons could inherit from their fathers [24]. On the other hand, frequent mating harms females in several ways, including genital trauma [47,48] and reduced foraging efficiency [17,19,49], especially in coercive mating systems [13,25]. Furthermore, sexual harassment can increase individuals' conspicuousness to predators [50–52]. An alternative, not mutually exclusive explanation of female preferences for sexually active males over control males could be social attraction. While fish in control animations only showed continuous swimming movement, animations of sexually active males additionally showed nipping behaviour, which could attract focal females as it resembles conditions in natural shoals, or simply because the more active animation pair was more conspicuous. However, those explanations fail to explain the observed bell-shaped preference function, especially the distinct (context-dependent) avoidance of animations showing the highest level of sexual activity. Avoidance of male sexual harassment provides a more plausible explanation for this pattern.

Female mosquitofish avoid male sexual harassment by forming all-female shoals, in which male harassment is diluted and females benefit from increased group vigilance [17,18]. Thus, shoaling lowers the costs of mating with highly sexually active males. Indeed, females changed their behaviour when exerting mate choice in the vicinity of three conspecific females: the distinct avoidance of highly sexually active males was no longer detectable. Our results, therefore, confirm that female preferences for male sexual activity are dependent on social context. In natural populations, poeciliids face a highly variable social environment due to temporal and spatial fluctuations in population densities [52,53], sex ratios [54,55] and group cohesiveness [56]. Accordingly, context-dependent adjustment of female mate choice could indeed contribute to the maintenance of consistent individual differences in sexual activity among males in natural populations.

Also in other poecillids, direct costs of mating with high-quality males can lead to patterns of context-dependent female mate choice. For example, male ornamentation in guppies (*Poecilia reticulata*) is an indicator of male quality [57–60] and females prefer brightly coloured males [52]. In the presence of predators, however, females prefer paler and less conspicuous males [61–63], probably to decrease their own risk of falling victim to predation [51,64]. Likewise, *Poecilia mexicana* females show a predilection for large, colourful males but prefer smaller, less colourful ones in the presence of predators [65].

Finally, we asked if the pattern reported here is learned, or part of the innate behavioural repertoire of our study species. In our study, females that never interacted with males before the mate choice tests did not show any preference related to male sexual activity. This finding has two important implications: (i) virgin females potentially also contribute to the maintenance of variation in male sexual activity and (ii) female choice for male sexual activity requires sexual experience. Given that poeciliid fishes were repeatedly shown to observe sexual interactions in their social environment (e.g. during mate choice copying [27,66,67]), an interesting question for future studies would be whether females learn only from own sexual interactions or whether (and to what extent) they might also use social information.

## Supplementary Material

Consistent individual differences in male sexual activity; Separation of premature males and females; Experimental setup for the assessment of mating preferences; Table S5; Additional references

## Data accessibility

All data are accessible as additional spread sheets in the electronic supplementary material.

## References

[1] Andersson M 1994 Sexual selection. Princeton, NJ: Princeton University Press.

[2] Packer C 1979 Male dominance and reproductive activity in *Papio anubis*. Anim. Behav. 27, 37–45. (doi:10.1016/0003-3472(79)90127-1) 555842 10.1016/0003-3472(79)90127-1

[3] Widemo F, Owen IPF 1995 Lek size, male mating skew and the evolution of lekking. Lett. Nat. 373, 148–151. (doi:10.1038/373148a0)

[4] Rubenstein DR, Lovette IJ 2009 Reproductive skew and selection on female ornamentation in social species. Nature 462, 786–789. (doi:10.1038/nature08614) 20010686 10.1038/nature08614

[5] Emlen ST, Oring LW 1977 Ecology, sexual selection and the evolution of mating systems. Science 197, 215–223. (doi:10.1126/science.327542) 327542 10.1126/science.327542

[6] Forbes MRL 1991 Ectoparasites and mating success of male *Enallagma erbium* damselflies (Odonata: Coenagrionidae). Oikos 60, 336–342. (doi:10.2307/3545076)

[7] Gustafsson L, Nordling D, Andersson MS, Sheldon BC, Qvarnström A 1997 Infectious diseases, reproductive effort and the cost of reproduction in birds. Phil. Trans. R. Soc. Lond. B 346, 323–331. (doi:10.1098/rstb.1994.0149) 10.1098/rstb.1994.0149 7708827

[8] Mappes J, Alatalo RV, Kotiaho J, Parri S 1996 Viability costs of condition-dependent sexual male display in a drumming wolf spider. Proc. R. Soc. Lond. B 263, 785–789. (doi:10.1098/rspb.1996.0117)

[9] Abrahams MV, Robb TL, Hare JF 2005 Effect of hypoxia on opercular displays: evidence for an honest signal. Anim. Behav. 70, 427–432. (doi:10.1016/j.anbehav.2004.12.007)

[10] Magellan K, Magurran AE 2007 Behavioural profiles: individual consistency in male mating behaviour under varying sex ratios. Anim. Behav. 74, 1545–1550. (doi:10.1016/j.anbehav.2007.03.015)

[11] Godin J-GJ, Auld HL 2013 Covariation and repeatability of male mating effort and mating preferences in a promiscuous fish. Ecol. Evol. 3, 2020–2029. (doi:10.1002/ece3.607) 23919148 10.1002/ece3.607 PMC3728943

[12] Bisazza A 1993 Male competition, female mate choice and sexual size dimorphism in poeciliid fishes. In Behavioural ecology of fishes. (eds Huntingford FA, Torricelli P). Chur, Switzerland: Harwood Academic.

[13] Pyke GH 2005 A review of the biology of *Gambusia affinis* and *G. holbrooki*. Rev. Fish Biol. Fisher. 15, 339–365. (doi:10.1007/s11160-006-6394-x)

[14] Reále D, Boussès P, Chapuis J-L 1996 Female-biased mortality induced by male sexual harassment in a feral sheep population. Can. J. Zool. 74, 1812–1818. (doi:10.1139/z96-202)

[15] McKinney F, Evarts S 1997 Sexual coercion in waterfowl and other birds. Ornithol. Monogr. 49, 163–195.

[16] Gay L, Eady PE, Vasudev R, Hosken DJ, Tregenza T 2009 Costly sexual harassment in a beetle. Physiol. Entomol. 34, 86–92. (doi:10.1111/j.1365-3032.2008.00656.x)

[17] Pilastro A, Benetton S, Bisazza A 2003 Female aggregation and male competition reduce costs of sexual harassment in the mosquitofish *Gambusia holbrooki*. Anim. Behav. 65, 1161–1167. (doi:10.1006/anbe.2003.2118)

[18] Dadda M, Pilastro A, Bisazza A 2005 Male sexual harassment and female schooling behaviour in the eastern mosquitofish. Anim. Behav. 70, 463–471. (doi:10.1016/j.anbehav.2004.12.010)

[19] Magurran AE, Seghers BH 1994 A cost of sexual harassment in the guppy, *Poecilia reticulata*. Proc. R. Soc. Lond. B 258, 89–92. (doi:10.1098/rspb.1994.0147)

[20] Plath M, Makowicz A, Schlupp I, Tobler M 2007 Sexual harassment in live-bearing fishes (Poeciliidae): comparing courting and noncourting species. Behav. Ecol. 18, 680–688. (doi:10.1093/beheco/arm030)

[21] Bierbach D, Jung CT, Hornung S, Streit B, Plath M 2013 Homosexual behaviour increases male attractiveness to females. Biol. Lett. 9, 20121038 (doi:10.1098/rsbl.2012.1038) 23234866 10.1098/rsbl.2012.1038 PMC3565526

[22] Kodric-Brown A 1985 Female preference and sexual selection for male coloration in the guppy (*Poecilia reticulata*). Behav. Ecol. Sociobiol. 17, 199–205. (doi:10.1007/BF00300137)

[23] McPeek M 1992 Mechanisms of sexual selection operating on body size in the mosquitofish (*Gambusia holbrooki*). Behav. Ecol. 3, 1–12. (doi:10.1093/beheco/3.1.1)

[24] Weatherhead PJ, Robertson RJ 1997 Offspring quality and the polygyny threshold: ‘the sexy son hypothesis’. Am. Nat. 113, 201–208. (doi:10.1086/283379)

[25] Bisazza A, Marin G 1995 Sexual selection and sexual size dimorphism in the eastern mosquitofish *Gambusia holbrooki* (Pisces: Poeciliidae). Ethol. Ecol. Evol. 7, 169–183. (doi:10.1080/08927014.1995.9522963)

[26] Krause J, Ruxton GD. 2002 Living in groups. Oxford, UK: Oxford University Press.

[27] Dugatkin LA 1996 Interface between culturally based preferences and genetic preferences: female mate choice in *Poecilia reticulata*. Proc. Natl Acad. Sci. USA 93, 2770–2773. (doi:10.1073/pnas.93.7.2770) 11607646 10.1073/pnas.93.7.2770 PMC39707

[28] Farr JA 1989 Sexual selection and secondary differentiation in poeciliids: determinants of male mating success and the evolution of female choice. In Ecology and evolution of livebearing fishes (Poeciliidae) (eds Meffe GK, Snelson FF), pp. 91–123. Englewood Cliffs, NJ: Prentice Hall.

[29] Pilastro A, Giacomello E, Bisazza A 1997 Sexual selection for small size in male mosquitofish (*Gambusia holbrooki*). Proc. R. Soc. Lond. B 264, 1125–1129. (doi:10.1098/rspb.1997.0155)

[30] Pilastro A, Bisazza A 1999 Insemination efficiency of two alternative male mating tactics in the guppy (*Poecilia reticulata*). Proc. R. Soc. Lond. B 266, 1887–1891. (doi:10.1098/rspb.1999.0862)

[31] Bisazza A, Vaccari G, Pilastro A 2001 Female mate choice in a mating system dominated by male sexual coercion. Behav. Ecol. 12, 59–64. (doi:10.1093/oxfordjournals.beheco.a000379)

[32] Busack CA, Gall GAE 1983 An initial description of the quantitative genetics of growth and reproduction in the mosquitofish, *Gambusia affinis*. Aquaculture 32, 123–140. (doi:10.1016/0044-8486(83)90275-2)

[33] Evans JP, Pierotti M, Pilastro A 2003 Male mating behavior and ejaculate expenditure under sperm competition risk in the eastern mosquitofish. Behav. Ecol. 14, 168–273. (doi:10.1093/beheco/14.2.268)

[34] Franck D, Geissler U 1973 Versuche zur Veränderung der sexuellen Handlungsbereitschaft nach kurzfristiger sozialer Isolation bei *Xiphophorus helleri*. Ethology 33, 408–416. 4785209

[35] Franck D 1975 Der Anteil des ‘Coolidge-Effektes’ an der isolationsbedingten Zunahme sexueller Verhaltensweisen von *Poecilia sphenops*. Z. Tierpsychol. 38, 472–481. (doi:10.1111/j.1439-0310.1975.tb02020.x)

[36] Dewsbury DA 1981 Effects of novelty on copulatory behaviour: the Coolidge effect and related phenomena. Psychol. Bull. 89, 464–482. (doi:10.1037/0033-2909.89.3.464)

[37] Kelley JL, Graves JA, Magurran AE 1999 Familiarity breeds contempt in guppies. Nature 401, 661–662. (doi:10.1038/44314) 10537103 10.1038/44314

[38] Agrillo C, Dadda M, Serena G, Bisazza A 2008 Do fish count? Spontaneous discrimination of quantity in female mosquitofish. Anim. Cogn. 11, 495–503. (doi:10.1007/s10071-008-0140-9) 18247068 10.1007/s10071-008-0140-9

[39] Bischoff RJ, Gould JL, Rubenstein DI 1985 Tail size and female choice in the guppy (*Poecilia reticulata*). Behav. Ecol. Sociobiol. 46, 169–175. (doi:10.1007/bf00300143)

[40] Kodric-Brown A 1993 Female choice of multiple male criteria in guppies: interacting effects of dominance, coloration and courtship. Behav. Ecol. Sociobiol. 32, 415–420.

[41] Walling CA, Royle NJ, Lindström J, Metcalfe NB 2010 Do female association preferences predict the likelihood of reproduction? Behav. Ecol. Sociobiol. 65, 541–548. (doi:10.1007/s00265-009-0869-4)

[42] Chouinard-Thuly L et al. 2017 Technical and conceptual considerations for using animated stimuli in studies of animal behaviour. Curr. Zool. 63, 5–19. (doi:10.1093/cz/zow104) 10.1093/cz/zow104 PMC5804155 29491958

[43] Rosentahl GG, Evans CS 1998 Female preference for swords in *Xiphophorus helleri* reflects a bias for large apparent size. Proc. Natl Acad. Sci. USA 95, 4431–4436. (doi:10.1073/pnas.95.8.4431) 9539754 10.1073/pnas.95.8.4431 PMC22506

[44] Kodric-Brown A, Nicoletto PF 2001 Female choice in the guppy (*Poecilia reticulata*): the interaction between male color and display. Behav. Ecol. Sociobiol. 50, 346–351. (doi:10.1007/s002650100374)

[45] Fisher HS, Mascuch SJ, Rosenthal GG 2009 Multivariate male traits misalign with multivariate female preferences in the swordtail fish, *Xiphophorus birchmanni*. Anim. Behav. 78, 265–269. (doi:10.1016/j.anbehav.2009.02.029)

[46] Polverino G, Liao JC, Porfiri M. 2013 Mosquitofish (*Gambusia affinis*) preference and behavioural response to animated images of conspecifics altered in their color, aspect ratio, and swimming depth. PLoS ONE 8, e54315 (doi:10.1371/journal.pone.0054315) 23342131 10.1371/journal.pone.0054315 PMC3546983

[47] Peters G, Mäder B 1964 Morphologische Veränderungen der Gonadenausführgänge sich fortpflanzender Schwertträgerweibchen (*Xiphophorus helleri* Heckel). Zool. Anz. 173, 243–257.

[48] Greven H, Uribe MC, Grier HJ. 2005 Structural and behavioural traits associated with sperm transfer. In *Poeciliinae, Viviparous fishes*, pp. 145–163. Homestead, FL: New Life Publications.

[49] Köhler A, Hildebrand P, Schleucher E, Riesch R, Arias-Rodriguez L, Streit B, Plath M 2011 Effects of male sexual harassment on female time budgets, feeding behaviour, and metabolic rates in a tropical livebearing fish (*Poecilia mexicana*). Behav. Ecol. Sociobiol. 65, 1513–1523. (doi:10.1007/s00265-011-1161-y)

[50] Magurran AE, Seghers BH 1994 Sexual conflict as a consequence of ecology: evidence from guppy, *Poecilia reticulata*, populations in Trinidad. Proc. R. Soc. Lond. B 255, 31–36. (doi:10.1098/rspb.1994.0005)

[51] Pocklington R, Dill LM 1995 Predation on females or males: who pays for bright male traits? Anim. Behav. 49, 1122–1124. (doi:10.1006/anbe.1995.0141)

[52] Houde AE. 1997 Sex, color, and mate choice in guppies. Princeton, NJ: Priceton University Press.

[53] Chapman LJ, Kramer DL, Chapman CA 1991 Population dynamics of the fish *Poecilia gillii* (Poeciliidae) in pools of an intermittent tropical stream. J. Anim. Ecol. 60, 441–453. (doi:10.2307/5289)

[54] Britton RH, Moser ME 1982 Size specific predation by herons and its effect on the sex ratio of natural populations of the mosquitofish *Gambusia affinis* (Baird and Girard). Oecologia 53, 146–151. (doi:10.1007/BF00545657) 28311103 10.1007/BF00545657

[55] Pettersson LB, Ramnarine IW, Becher SA, Mahabir R, Magurran AE 2004 Sex ratio dynamics and fluctuating selection pressures in natural populations of the Trinidadian guppy, *Poecilia reticulata*. Behav. Ecol. Sociobiol. 55, 461–468. (doi:10.1007/s00265-003-0727-8)

[56] Kimbell HS, Morrell LJ 2015 Turbidity influences individual and group level responses to predation in guppies, *Poecilia reticulata*. Anim. Behav. 103, 179–185. (doi:10.1016/j.anbehav.2015.02.027)

[57] Nicoletto PF 1991 The relationship between male ornamentation and swimming performance in the guppy, *Poecilia reticulata*. Behav. Ecol. Sociobiol. 28, 365–370. (doi:10.1007/BF00164386)

[58] Houde AE, Torio AJ 1992 Effect of parasitic infection on male color pattern and female choice in guppies. Behav. Ecol. 3, 346–351. (doi:10.1093/beheco/3.4.346)

[59] Grether GF, Hudon J, Millie DF 1999 Carotenoid limitation of sexual coloration along an environmental gradient in guppies. Proc. R. Soc. Lond. B 266, 1317–1322. (doi:10.1098/rspb.1999.0781)

[60] Locatello L, Rasotto MB, Evans JP, Pilastro A 2006 Colourful male guppies produce faster and more viable sperm. J. Evol. Biol. 19, 1595–1602. (doi:10.1111/j.1420-9101.2006.01117.x) 16910988 10.1111/j.1420-9101.2006.01117.x

[61] Breden F, Stoner G 1987 Male predation risk determines female preference in the Trinidad guppy. Nature 329, 831–833. (doi:10.1038/329831a0)

[62] Godin J-GJ, Briggs SE 1996 Female mate choice under predation risk in the guppy. Anim. Behav. 51, 117–130. (doi:10.1006/anbe.1996.0010)

[63] Gong A, Gibson RM 1996 Reversal of a female preference after visual exposure to a predator in the guppy, *Poecilia reticulata*. Anim. Behav. 52, 1007–1015. (doi:10.1006/anbe.1996.0248)

[64] Godin J-GJ, McDonough HE 2003 Predator preference for brightly colored males in the guppy: a viability cost for a sexually selected trait. Behav. Ecol. 14, 194–200. (doi:10.1093/beheco/14.2.194)

[65] Bierbach D et al. 2011 Predator-induced changes of female mating preferences: innate and experiential effects. BMC Evol. Biol. 11, 190 (doi:10.1186/1471-2148-11-190) 21726456 10.1186/1471-2148-11-190 PMC3141438

[66] Dugatkin LA, Godin J-GJ 1992 Reversal of female mate choice by copying in the guppy (*Poecilia reticulata*). Proc. R. Soc. Lond. B 249, 179–184. (doi:10.1098/rspb.1992.0101) 10.1098/rspb.1992.0101 1360679

[67] Witte K, Ryan MJ 2002 Mate choice copying in the sailfin molly, *Poecilia latipinna*, in the wild. Anim. Behav. 63, 943–949. (doi:10.1006/anbe.2001.1982)

