# Supplementary material for: Context-dependent female mate choice maintains variation in male sexual activity
Source: R Soc Open Sci. 2017 Jul 12;4(7):170303. doi: 10.1098/rsos.170303 (PMC5541552; doi:10.1098/rsos.170303)
Supplement: Consistent individual differences in male sexual activity; Separation of premature males and females; Experimental setup for the assessment of mating preferences; Table S5; Additional references [file rsos170303supp1.docx]

**Supplementary material**

**S1 Consistent individual differences in male sexual activity**

**Background**

Individual differences in male mating activity can reflect consistent differences in intrinsic male mating activity [1,2]. Similar patterns of behavioural traits that defy optimal response theories by appearing constrained in plasticity are target of the rising field of studies on animal personality [3]. Individuals differ consistently in personality traits like boldness [4], aggressiveness [5], or activity [6], but also correlations between different traits (so-called behavioural syndromes) are frequently observed [7-9]. Here, we provide additional data demonstrating pronounced individual variation, coupled with high behavioural consistency, in male sexual activity of our study species.

In theory male sexual activity could be part of a behavioural syndrome (i.e., activity syndrome). We, therefore, also considered locomotor activity (swimming activity), one of the most studied personality traits [3] that was shown to be highly repeatable in poeciliid fishes, including *Gambusia affinis* [10], *Poecilia mexicana* [6], and guppies (*Poecilia reticulata*) [11]. We first tested whether *G. holbrooki* males differ consistently in individual sexual activity in absence of intrasexual competition and with ample mating opportunities. Second, we tested the same males for consistency in swimming activity and asked if both traits are correlated.

**Methods**

Test fish were laboratory-reared descendants of wild-caught individuals collected in the species’ invasive range in Torre Castiglione (Puglia, Italy) in summer 2014. To test for consistent individual differences in male sexual activity, we randomly assigned one male per trial to a group of three unfamiliar females (mean ± SE standard length, SL: 38.2 ± 5.1 mm). Maintenance conditions in the six parallel test tanks (80 × 25 × 25 cm) were similar to those in the stock tanks (see Methods in the main article) but we did not provide stones and plants, such that females would not hide from males [12]. All sides except the front wall were covered with grey cardboard in order to reduce disturbance of the test fish and to block visual contact between different test tanks. After three days for acclimation, we conducted the first assessment of male sexual activity, which we repeated on three successive days to provide an estimate of behavioural repeatability [13]. We tested *n* = 18 males (SL: 26.3 ± 0.1 mm).

While recording male sexual activity, the experimenter was sitting quietly about 2 m in front of the test tank. For ten minutes, male sexual activity was recorded as the sum of the following behaviours: (*a*) ‘approaches’, where males approach a female in the blind portion of her visual field [14], (*b*) ‘nipping’, during which males touch the female’s genital pore with their snout [15], and (*c*) ‘gonopodial thrusts’, during which males turn the gonopodium (their copulatory organ, a modified anal fin) forward in an attempt to insert it into the female’s genital pore [14].

Second, we assessed swimming activity as the distance covered by a focal male within five minutes of observation. 24 hours after the last assessment of mating activity, we placed the focal male into a new test tank (80 × 25 × 20 cm) that was covered on all sides with grey cardboard and filled with aged tap water to a height of 10 cm. After the male was given 5 min to explore the tank, a camera installed 120 cm above the tank started recording swimming behaviour for another 5 min. To quantify swimming activity, a gridline (5 × 5 cm) was drawn on the bottom of the test tank. We later counted numbers of squares the male swam through, assuming that more active males would cover a larger number of squares than less active males [16-18]. Directly after the assessment of swimming activity, we measured the standard length (SL) of the focal males by laying them flat on moist laminated millimetre paper, after which they were retransferred into the original stock tank.

**Statistical analysis**

We asked whether males differ consistently in their sexual and locomotor activities and if both traits are correlated. We first ran separate linear mixed models (LMMs). For sexual activity, we included ‘test run’ (day) and ‘female group ID’ as fixed factors and ‘focal male SL’ as a covariate. For swimming activity, ‘focal male SL’ served as a covariate, and ‘test run’ (day) was included as a fixed factor. Note that ‘female group ID’ was not included in this model since swimming activity was recorded in a different test tank (without females). Using LMMs allowed us to estimate the total behavioural variance not accounted for by the fixed effects, which can be further decomposed into among- and within-individual variance components [19]. We also used these variance estimates to calculate repeatability estimates. The repeatability of behaviour is defined as the proportion of the behavioural variance attributable to differences among individuals [20]. A significant repeatability estimate is interpreted as evidence of consistent individual differences. To test for a correlation between both behavioural traits, we correlated average trait values using Spearman’s *ρ*.

**Results**

On average, males performed 2.0 ± 0.1 (mean ± SE) sexual behaviours per minute (range: 0.7 – 3.4 behaviours per minute). We found individual males to differ significantly in the magnitude of their intrinsic sexual activity, as indicated by high behavioural repeatability over the three days of repeated measurement (*R* = 0.59, 95% CI = 0.45–0.71; *p* < 0.001; figure S1). Neither ‘female group ID’ (*F*_5,11_ = 0.98, *p* = 0.47), ‘focal males’ body size’ (*F*_1,11_ = 1.54, *p* = 0.24), nor ‘test run’ (i.e., repeated testing; *F*_2,34_ = 1.28, *p* = 0.29) had statistically significant effects on male sexual activity in our LMM. When we considered only gonopodial thrusting instead (i.e., disregarding pre-copulatory behaviours) we found a similar repeatability estimate (*R* = 0.51, *p* < 0.001).

**
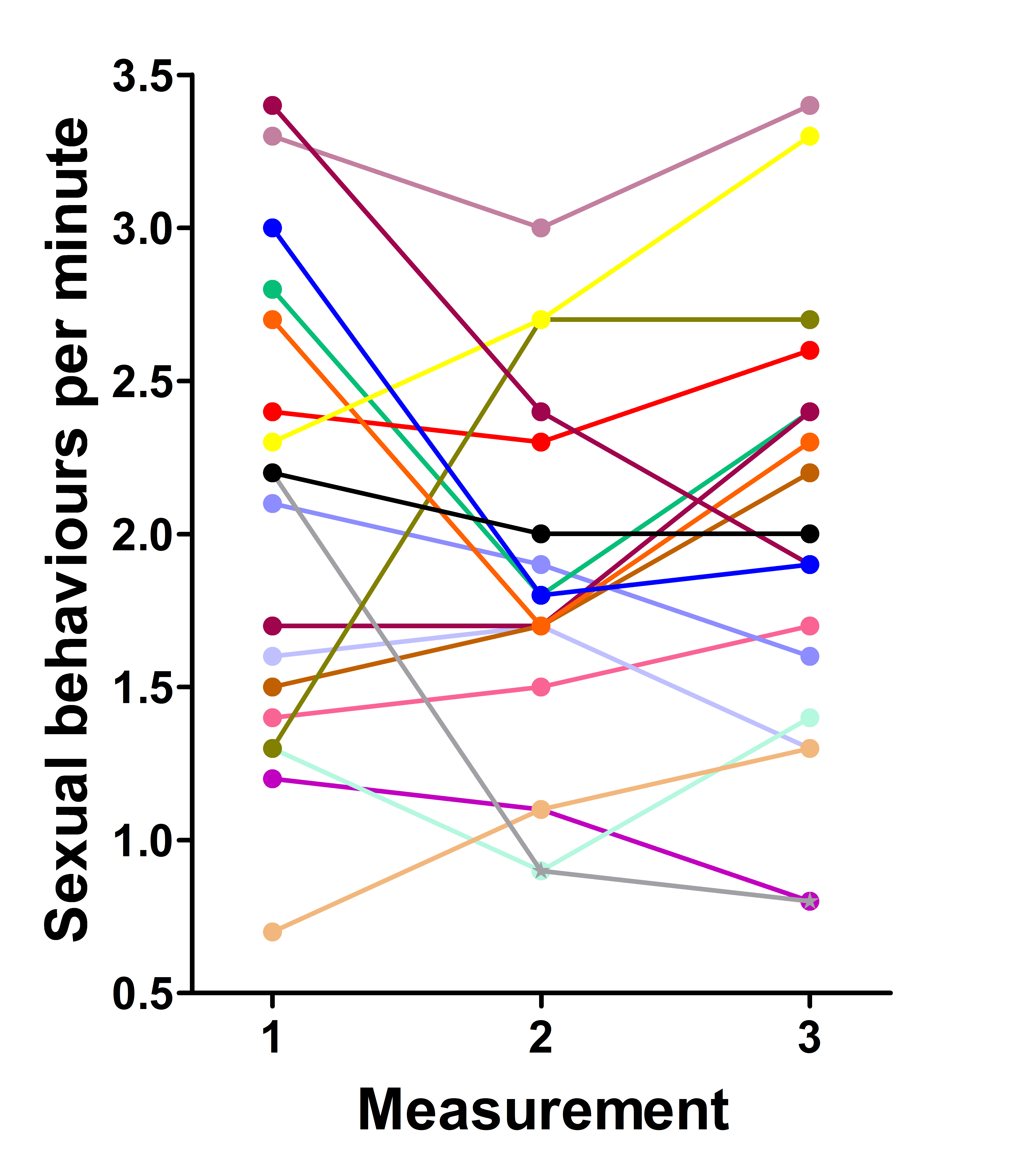
**

**Figure S1**

Sexual behaviours of 18 *G. holbrooki* males were quantified on three consecutive days. Each line and colour represents one individual, showing individual measurements of male sexual activity per minute. Pronounced among-individual differences (differences in intercepts) in combination with low within-individual variance (most lines have only moderate slopes) resulted in few lines crossing (i.e., rank orders remained similar over the course of measurements), which was reflected by high estimates of behavioural consistency/repeatability.

The same males also differed consistently in their swimming activity in an open field test (*R* = 0.65, 95% CI = 0.60–0.70; *p* = 0.001), but neither ‘male body size’ (LMM: *F*_1,16_ = 0.48, *p* = 0.50) nor ‘test run’ (*F*_1,17_ = 0.63, *p* = 0.44) had significant effects on swimming activity. Sexual activity and swimming activity were not correlated (Spearman correlation: *r*_S_ = 0.13, *p* = 0.62, *n* = 18), suggesting that, while both traits are highly repeatable, they are not part of a general activity syndrome.

**Discussion**

Variation in male sexual activity can be caused by extrinsic factors such as predation risk [21-23], infections [24,25], males’ position in dominance hierarchies [26], or fluctuation in mate competition due to different operational sex ratios, the latter being particularly well-documented for poeciliid fishes [27-29]. Here, we excluded those factors and provided all males with ample mating opportunities, but still found pronounced differences in male sexual activity. This is in line with studies on another poeciliid, the guppy, which reported on repeatable individual differences in male sexual effort [1], even under varying sex ratios [2]. In theory, this pattern could be explained by similar intrinsic (genetic) factors contributing to individual differences in both, sexual activity and locomotor activity, and selection on other components of this activity syndrome might maintain the observed behavioural polymorphism. Male sexual behaviour of *G. holbrooki* and several other poeciliids includes the frequent pursuit of females that are unwilling to mate [14]. Females try to avoid incessant sexual harassment by swimming away and seeking the vicinity of other females [14,30], and so male sexual behaviour could indeed be linked to general swimming activity. However, we did not find a correlation between locomotor and sexual activity in *G. holbrooki* males, rendering this explanation unlikely. Still, (epi-) genetic factors that affect male sexual behaviour could be involved in shaping consistent differences in male sexual activity, e.g., sexual steroids like testosterone and 11-ketotestosterone, which are known to regulate male mating behaviour in poeciliids [31,32] and other teleosts [33-35]. Titres of those hormones can indeed vary in a consistent manner among individuals [36-38].

In this experiment, we used a different population of *G. holbrooki* (from the species’ invasive range in Europe) than in our mate choice experiment. Invasive mosquitofish populations have lower genetic variation than populations in the species’ natural distribution range [39]. If this affected the results, it should have led to an underestimation of among-individual variation and thus, behavioural consistency. The fact that we still uncovered pronounced and consistent among-individual differences in male mating behaviour, therefore, supports the ideas outlined in the introduction of our main article.

**S2 Separation of premature males and females**

To obtain virgin (sexually naïve) females, it was crucial to separate males from females before they reached sexual maturity. Poeciliid fishes have internal fertilization, for which males insert their gonopodium—a modified anal fin through which sperm bundles are being transferred—into the female’s gonopore [40].

The metamorphosis of the gonopodium takes several weeks and in the related *G. affinis* is usually completed by the age of 120-180 days [41]. During metamorphosis, anal fin rays 3, 4, and 5 are elongated by addition of segments until they become more than twice as long as the other anal fin rays. At the same time, anal fin ray 3 becomes thicker by the addition of new bone material [41,42]. Sex differences in anal fin morphology become visible early (for more details see [41]), which allowed us to separate males from females before their gonopodium reached a stage in which copulations became possible.

**S3 Experimental setup for the assessment of mating preferences**


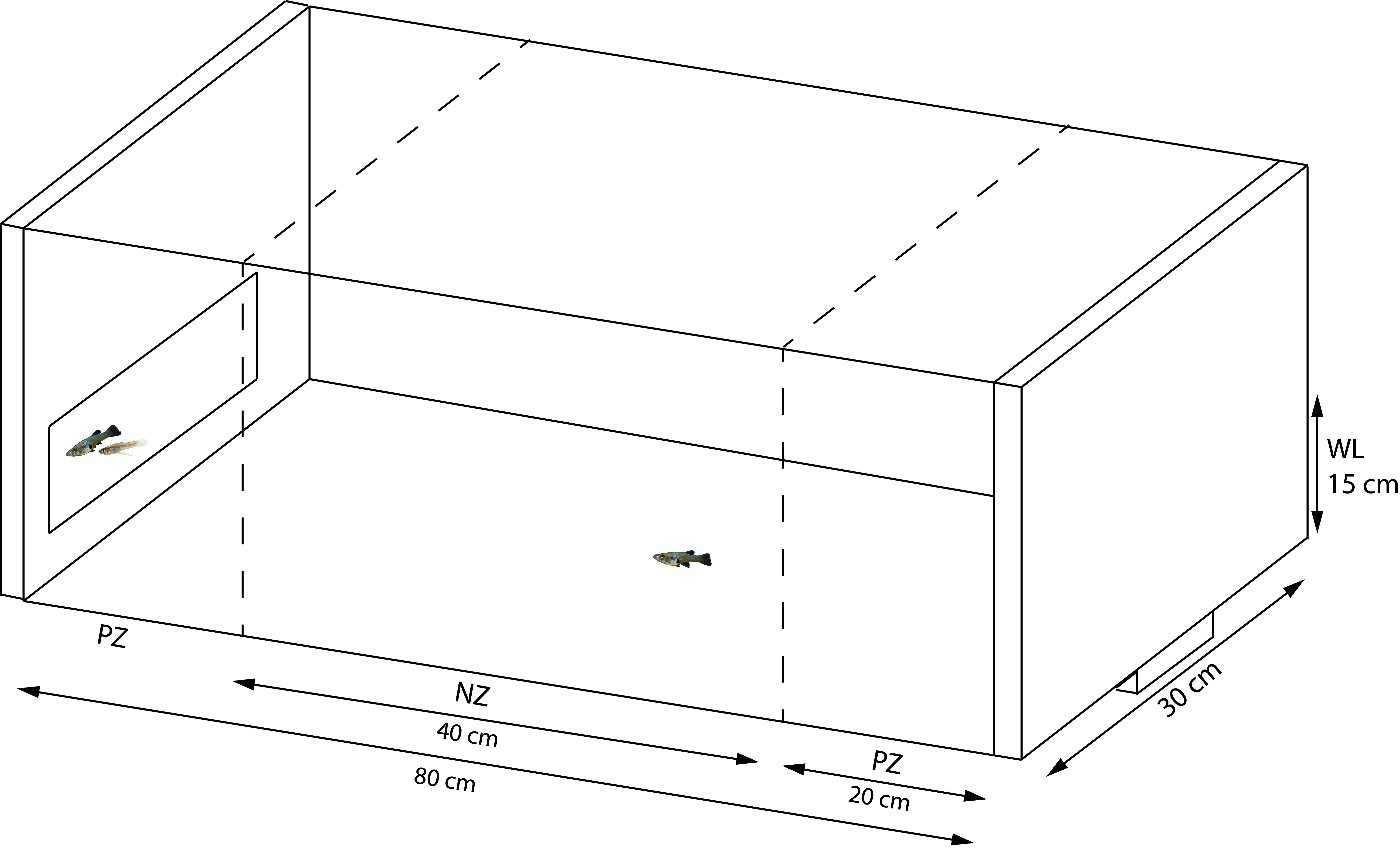


**Figure S3**

Schematic view of the experimental set-up used in the mate choice trials. The test tank was visually divided into a neutral (NZ, centre) and two lateral preference zones (PZ). Two adjacent screens on both ends of the tank showed animated stimulus males (i.e., sexual interactions with a virtual female on one side and no sexual interactions on the other side).

**S4**

Example video of an animated stimulus male showing sexual interactions (‘nipping’ behaviour) with a virtual female at an intermediate frequency (3 sexual behaviours per minute).

**S5**

**Table S5**

Estimated marginal means from a linear mixed model using females’ strength of preference (SOP) for (*a*) low, (*b*) medium, and (*c*) high male sexual activity as the dependent variable, along with corresponding 95% confidence intervals (CI).

|  |  | **95% Confidence intervals** | |
| --- | --- | --- | --- |
| **Treatment** | **Mean** | **Lower CI** | **Upper CI** |
| **(*a*) Low male sexual activity** | | | |
| Experienced | 0.503 | 0.412 | 0.594 |
| Group | 0.523 | 0.432 | 0.614 |
| Virgin | 0.450 | 0.367 | 0.533 |
| **(*b*) Medium male sexual activity** | | | |
| Experienced | 0.710 | 0.619 | 0.801 |
| Group | 0.640 | 0.549 | 0.731 |
| Virgin | 0.514 | 0.431 | 0.597 |
| **(*c*) High male sexual activity** | | | |
| Experienced | 0.339 | 0.248 | 0.430 |
| Group | 0.586 | 0.495 | 0.677 |
| Virgin | 0.543 | 0.460 | 0.626 |

**Additional references**

1. Godin J-GJ, Auld HL. 2013 Covariation and repeatability of male mating effort and mating preferences in a promiscuous fish. *Ecol. Evol.* **3**, 2020-2029.

2. Magellan K, Magurran AE. 2007 Behavioural profiles: individual consistency in male mating behaviour under varying sex ratios. *Anim. Behav.* **74**, 1545-1550.

3. Réale D, Reader SM, Sol D, McDougall PT, Dingemanse NJ. 2007 Integrating animal temperament within ecology and evolution. *Biol. Rev.* **82**, 291-318.

4. Wilson ADM, Godin J-GJ. 2009 Boldness and behavioural syndromes in the bluegill sunfish, *Lepomis macrochirus*. *Behav. Ecol.* **20**, 231-237.

5. Blumstein DT, Petelle MB, Wey TW. 2013 Defensive and social aggression: repeatable but independent. *Behav. Ecol.* **24**, 457-461.

6. Sommer-Trembo C, Bierbach D, Arias-Rodriguez L, Verel Y, Jourdan J, Zimmer C, Riesch R, Streit B, Plath M. 2016 Does personality affect premating isolation between locally-adapted populations? *BMC Evol. Biol*.: DOI 10.1186/s12862-016-0712-2.

7. Sih A, Bell A, Johnson C. 2004 Behavioral syndromes: an ecological and evolutionary overview. *Trends. Ecol. Evol.* **19**, 372-378.

8. Duckworth RA. 2006 Behavioral correlations across breeding contexts provide a mechanism for a cost of aggression. *Behav. Ecol.* **17**, 1011-1019.

9. Dingemanse NJ, Wright J, Kazem AJN, Thomas DK, Hickling R, Dawnay N. 2007 Behavioural syndromes differ predictably between 12 populations of three-spined stickleback. *J. Anim. Ecol.* **76**, 1128-1138.

10. Cote J, Fogarty S, Weinersmith K, Brodin T, Sih A. 2010 Personality traits and dispersal tendency in the invasive mosquitofish (*Gambusia affinis*). *Proc. R. Soc. Lond. B*. **277**, 1571-1579.

11. Brown C, Irving E. 2013 Individual personality traits influence group exploration in a feral guppy population. *Behav. Ecol.* **25**, 95-101.

12. Bisazza A. 1993 Male competition, female mate choice and sexual size dimorphism in poeciliid fishes. In: Behavioural ecology of fishes. Huntingford FA, Torricelli P (eds.). pp 257-286. Chur: Harwood Academic.

13. Bell A, Hankison SJ, Laskowski KL. 2009 The repeatability of behaviour: a meta-analysis. *Anim. Behav*. **77**, 771-783.

14. Pilastro A, Benetton S, Bisazza A. 2003 Female aggregation and male competition reduce costs of sexual harassment in the mosquitofish *Gambusia holbrooki*. *Anim. Behav*. **65**, 1161-1167.

15. Plath M, Makowicz A, Schlupp I, Tobler M. 2007 Sexual harassment in live-bearing fishes (Poeciliidae): comparing courting and noncourting species. *Behav. Ecol.* **18**, 680-688.

16. Burns JG. 2008 The validity of three tests of temperament in guppies (*Poecilia reticulata*). *J. Comp. Psychol.* **122**: 344.

17. Archard GA, Braithwaite VA. 2011 Increased exposure to predators increases both exploration and activity level in *Brachyrhaphis episcopi*. *J. Fish. Biol.* **78**, 593-601.

18. Bierbach D, Sommer-Trembo C, Hanisch J, Wolf M, Plath M. 2015 Personality affects mate choice: bolder males show stronger audience effects under high competition. *Behav. Ecol.* **26**, 1314-1325.

19. Dingemanse NJ, Dochtermann NA. 2013 Quantifying individual variation in behaviour: mixed modelling approaches. *J. Anim. Ecol*. **82**, 39-54.

20. Nakagawa S, Schielzeth H. (2010) Repeatability for Gaussian and non-Gaussian data: a practical guide for biologists. *Biol. Rev.* **85**, 935-956.

21. Farr JA. 1975 The role of predation in the evolution of social behavior of natural populations of the guppy, *Poecilia reticulata* (Pisces: Poeciliidae). *Evolution* **29**, 151-158.

22. Endler JA. 1987 Predation, light intensity and courtship behaviour in *Poecilia reticulata* (Pisces: Poeciliidae). *Anim. Behav.* **35**, 1376-1385.

23. Sih A. 1988 The effects of predators on habitat use, activity, and mating behaviour of a semi-aquatic bug. *Anim. Behav.* **36**, 1846-1848.

24. Morales J, Larralde C, Arteaga M, Govezensky T, Romano MC, Morali G. 1969 Inhibition of sexual behavior in male mice infected with *Taenia crassiceps cysticerci*. *J. Parasitol*. **82**, 689-693.

25. Kolluru GR, Grether GF, Dunlop E, South SH. 2009 Food availability and parasite infection influence mating tactics in the guppy (*Poecilia reticulata*). *Behav. Ecol.* **20**, 131-137.

26. Le Boeuf BJ, Peterson RS. 1969 Social status and mating activity in elephant seals. *Science* **163**, 91-93.

27. Evans JP, Magurran AE. 1999 Male mating behaviour and sperm production characteristics under varying sperm competition risk in guppies. *Anim. Behav.* **58**, 1001-1006.

28. Evans JP, Pierotti M, Pilastro A. 2003 Male mating behaviour and ejaculate expenditure under sperm competition risk in the eastern mosquitofish. *Behav. Ecol.* **14**, 268-273.

29. Chuard PJC, Brown GE, Grant JWA. 2016 The effect of adult sex ratio on mating competition in male and female guppies (*Poecilia reticulata*) in two wild populations. *Behav. Proc*. **129**, 1-10.

30. Dadda M, Pilastro A, Bisazza A. 2005 Male sexual harassment and female schooling behaviour in the eastern mosquitofish. *Anim. Behav.* **70**, 463-471.

31. Toft G, Guillette LJ. 2005 Decreased sperm count and sexual behavior in mosquitofish exposed to water from a pesticide-contaminated lake. *Ecotoxicol. Environ. Saf.* **60**, 15-20.

32. Deaton R. 2008 Factors influencing male mating behaviour in *Gambusia affinis* (Baird & Girard) with a coercive mating system. *J. Fish. Biol*. **72**, 1607-1622.

33. Borg B. 1994 Androgens in teleost fishes. Comp. *Biochem. Physiol. C. Pharmacol. Toxicol. Endocrinol.* **109**, 219-245.

34. Pradhan A, Olsson P-E. 2015 Zebrafish sexual behaviour: role of sex steroid hormones and prostaglandins. *Behav. Brain. Funct*. **11.1** (2015): 1.

35. Sebire M, Katsiadaki I, Scott AP. 2007 Non-invasive measurement of 11-ketotestosterone, cortisol and androstenedione in male three-spined stickleback (*Gasterosteus aculeatus*). *Gen. Comp. Endocrinol*. **152**, 30-38.

36. Sellers JG, Mehl MR, Josephs RA. 2007 Hormones and personality: testosterone as a marker of individual differences. *J. Res. Personal*. **41**, 126-138.

37. Kralj-Fiser S, Scheiber IBR, Blejec A, Moestl E, Kotrschal K. 2007 Individualities in a flock of free-roaming greylag geese: behavioural and physiological consistency over time and across situations. *Horm. Behav*. **51**, 239-248.

38. van Oers K, Buchanan KL, Thomas TE, Drent PJ. 2011 Correlated response to selection of testosterone levels and immunocompetence in lines selected for avian personality. *Anim. Behav*. **81**, 1055-1061.

39. Grapputo A, Bisazza A, Pilastro A. 2006 Invasion success despite reduction of genetic diversity in the European populations of eastern mosquitofish (*Gambusia holbrooki*). *Ital. J. Zool*. **73**, 67-73.

40. Pyke GH. 2005 A review of the biology of *Gambusia affinis* and *G. holbrooki*. Rev. Fish. Biol. Fisher. **15**, 339-365.

41. Turner CL. 1941 Morphogenesis of the gonopodium in *Gambusia affinis affinis*. J. Morphol. **69**, 161-185.

42. Angus RA, McNatt HB, Howell WM, Peoples SD. 2001 Gonopodium development in normal male and 11-ketotestosterone-treated female mosquitofish (*Gambusia affinis*): a quantitative study using computer image analysis. Gen. Comp. Endocrinol. **123**, 222-234.
